# Recognizing Your Hand and That of Your Romantic Partner

**DOI:** 10.3390/ijerph17218256

**Published:** 2020-11-09

**Authors:** Takao Fukui, Aya Murayama, Asako Miura

**Affiliations:** Center for Applied Psychological Science, Kwansei Gakuin University, Nishinomiya 662-8501, Japan; ayamurayama@gmail.com (A.M.); asarin@hus.osaka-u.ac.jp (A.M.)

**Keywords:** hand recognition, self-other discrimination, social relationship

## Abstract

Although the hand is an important organ in interpersonal interactions, focusing on this body part explicitly is less common in daily life compared with the face. We investigated (i) whether a person’s recognition of their own hand is different from their recognition of another person’s hand (i.e., self hand vs. other’s hand) and (ii) whether a close social relationship affects hand recognition (i.e., a partner’s hand vs. an unknown person’s hand). For this aim, we ran an experiment in which participants took part in one of two discrimination tasks: (i) a self–others discrimination task or (ii) a partner/unknown opposite-sex person discrimination task. In these tasks, participants were presented with a hand image and asked to select one of two responses, self (partner) or other (unknown persons), as quickly and accurately as possible. We manipulated hand ownership (self (partner)/other(unknown person)), hand image laterality (right/left), and visual perspective of hand image (upright/upside-down). A main effect of hand ownership in both tasks (i.e., self vs. other and partner vs. unknown person) was found, indicating longer reaction times for self and partner images. The results suggest that close social relationships modulate hand recognition—namely, “self-expansion” to a romantic partner could occur at explicit visual hand recognition.

## 1. Introduction

Self–other discrimination is pivotal in social interaction (e.g., [1,2,3]). Face recognition plays a major role in distinguishing between self and other (e.g., [4]), whereas other body parts are assumed to play a less important role. However, neuroimaging studies have revealed that a specific brain area is selectively activated when perceiving body parts (i.e., the extrastriate body area, EBA) (e.g., [5,6,7]; see [8] for a review), in addition to the facial processing-specific fusiform face area [9]. The discovery of body-specific EBA has sparked increasing research interest regarding the ways in which body parts are perceived and recognized (e.g., [10,11,12]).

The hand is an undoubtedly crucial body part and serves as an important interface between the external world (including other persons) and the individual self. Researchers are interested in many aspects of the hand [13,14]. For example, the role of the hand has been investigated in non-verbal communication (e.g., [15]), motor behavior (e.g., [16]), and haptic perception (e.g., [17]). In computer vision studies, how a 3D hand structure could be estimated and reconstructed from a 2D image (e.g., [18,19]) has been investigated. In one neuroimaging study, Bracci, Ietswaart, Peelen, and Cavina-Pratesi [20] found a hand-preferring region in the EBA, suggesting that the representation of the hand in the extrastriate visual cortex is distinct from the representation of other body parts. Other researchers have found that the right EBA responds more to an allocentric view of body parts than to an egocentric view, and that this preference in the right EBA for the allocentric view could be linked with social cognition (i.e., self–other discrimination) [21,22]. In addition to these studies, the ways in which people visually distinguish between their own hands and the hands of others have been recently investigated, as discussed below.

Frassinetti and colleagues [11,23] reported a “self-advantage” effect in the visual matching-to-sample task, finding that self-related body stimuli are processed faster and more accurately than other-related body stimuli. Although the task used by Frassinetti et al. involved visual matching, it did not require the identification of hand ownership. Ferri, Frassinetti, Constantini, and Gallese [24] further investigated this effect using two tasks: one task involved hand laterality judgments of self hand and another’s hand, in which participants did not need to explicitly recognize the identity of the hand; the other task involved explicit self hand recognition. Ferri et al. [24] reported that the self-advantage effect emerged only when performing the laterality judgment, not when performing the explicit self-recognition task. Regarding the effects of visual perspective on an explicit self-recognition task, Conson, Aromino, and Trojano [25] reported that both right- and left-handed individuals were faster at recognizing their own dominant hands from an egocentric perspective and the other’s non-dominant hands from an allocentric perspective. 

Other persons can be classified from stranger to spouse in terms of “mental closeness” to oneself (i.e., familiarity, intimacy). For example, a romantic partner is regarded as a part of the self [26], and people indeed experience “self–partner confusion” concerning personality traits when they attempt to recognize them [26,27]. It has been found that face recognition is modulated by personal familiarity (e.g., [28,29]). The extent of interaction via the hands (e.g., touching another person’s body) changes according to the mental closeness of the individuals involved. Therefore, visual hand recognition could also be modulated by personal familiarity, although the personal identification of bodies would be assumed to be more difficult than that of faces. Answering this question could contribute to the further understanding of the mechanisms underlying self–other discrimination.

The aim of the current study was to investigate whether and how close relationships affect performance on an explicit hand discrimination task (cf. [30]). Specifically, we examined whether the explicit recognition of a partner’s hand is different from the explicit recognition of the hand of another person with whom the participant has no personal relationship. If we perform a hand laterality judgement task, deeper insight into the mechanisms of hand recognition will be provided. However, the current study included a “deceptive” procedure when taking photographs of hands, as described below. The hand laterality judgement task has to be performed before obtaining participants’ agreement for this deceptive procedure. Therefore, the hand laterality judgement task was not performed primarily for ethical reasons. 

## 2. Materials and Methods 

### 2.1. Participants

Participants took part in either (i) the self/other discrimination task, in which the participants were required to respond (i.e., self or other) to the presented hand stimulus (Task 1), or (ii) the partner/unknown opposite-sex person discrimination task, in which the participants were required to respond (i.e., partner or unknown opposite-sex person) to the presented hand stimulus (Task 2). A group of 23 self-reported right-handed students participated in the self/other discrimination task (Task 1), and a group of 20 self-reported right-handed students participated in the partner/unknown opposite-sex person discrimination task (Task 2). In accordance with the aim of this study, we excluded participants who noticed that photographs of their hands were being taken (see stimuli and procedure section for details) from the final analysis. Thus, the data of 18 students (mean age = 21.7 ± 2.7 years, 10 females) in the self/other discrimination task and 15 students (mean age = 21.7 ± 2.4 years, seven females) in the partner/unknown opposite-sex person discrimination task were included in the final analysis. All the participants reported normal or corrected-to-normal vision, and none of the participants had any motor or sensory abnormalities. No participants were homosexual based on self-reports. Concerning Task 2, the participants were not required to report the length of their relationship with their partner. The participants gave written informed consent to participate in the study, which was approved by the Kwansei Gakuin University Research Ethics Committee (no. 2011-24) according to the Declaration of Helsinki.

### 2.2. Stimuli and Procedure

#### 2.2.1. Photographing Participants’ Hands

##### Task 1: Self–Other Discrimination Task

Photographing the participants’ hands was followed by the hand discrimination task. To avoid the participants paying explicit attention to their own hands before the task, we sought to take photographs of the participants’ hands without them noticing. During recruitment, the participants were told that the experiment consisted of two parts: first, recording facial expressions by video camera, and second, a face recognition task. Before the camera recording, the participants were required to maintain a particular posture for several minutes as a “deceptive” baseline condition. Specifically, the participants sat comfortably on a chair and placed their hands in a box placed on a table. The palm was touching the bottom of the box and a camera was attached to the ceiling of the box. To avoid clues that could have led to the participants becoming aware that their hands were being photographed (e.g., the sound and/or flash of the camera), they were required to wear headphones and an eye mask. After this “secret” photographing of the backs of the participants’ hands, the recording of facial expressions (i.e., the fake task) was performed. 

**Figure 1:**
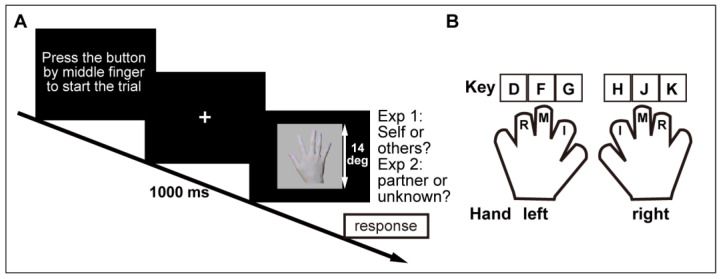
Experimental protocol. (**A**) Time sequence of the trial. (**B**) Finger configuration for key response. I, M, R denote index, middle, and ring fingers, respectively.

Stimuli were full-color photos of the left and right hands of participants, viewed from the back in a neutral, frontal, upright posture against a non-reflecting gray background (see Figure 1A). The original images of the hands (one picture per hand) in an upright orientation (0 deg, consistent with the perspective of the viewer—i.e., upright) were digitally manipulated to obtain hand images in the opposite orientation (180 deg—i.e., upside-down) (cf. [25]). Hand stimuli were edited by the Adobe Photoshop software.

##### Task 2: Partner/Unknown Opposite-Sex Person Discrimination Task

The hands of the partner of each participant for this task were photographed in the same way as mentioned above.

#### 2.2.2. Hand Discrimination Task

##### Task 1: Self–Other Discrimination Task

The participants were required to rest their fingers on a keyboard in the following position: index, middle, and ring fingers of the right hand were assigned to the H, J, and K keys, respectively, and the corresponding fingers of the left hand were assigned to the G, F, and D keys, respectively (see Figure 1B). An experimenter then hid the participants’ hands and keyboard from view by covering them with a sheet of paper, after which the actual purpose of the camera recording session was explained. After obtaining the participants’ agreement about this experimental procedure, the discrimination task commenced. 

The participants viewed the CRT display (60 Hz) from a distance of 57 cm, with their head movement restricted by a chin-rest. The experimental session consisted of four blocks (see below), and the response hand (right or left hand) was fixed in each block and ordered in an ABBA manner. The order (i.e., right/left/left/right or left/right/right/left) was counterbalanced across the participants. In each block, one of the index and ring fingers was assigned to responding to the participant’s own hand images, and the other was assigned to responding to images of another person’s hand. That is, the participants only used two fingers in one hand for the response in each block. This response finger pattern (i.e., index for own image and ring for another person’s image, or index for another person’s image and ring for own image) was counterbalanced across the participants. At the beginning of a trial, the participants were required to press J or F using the middle finger of either hand. A fixation cross was presented 1000 ms after pressing the J or F. A hand stimulus (14 deg × 14 deg) was presented until the participants responded (see Figure 1A). The participants were instructed to respond as quickly and accurately as possible. The task was to decide whether the presented hand stimulus was an image of their own hand or another person’s hand, and to press one of two keys (i.e., H (G) or K (D)). 

Two photographs of each hand of each participant were used as their own hand images, and four photographs from two different persons (same sex as the participant) whom the participant did not know were used as the others’ hand stimuli (i.e., 2 pictures × 2 persons). The four conditions (upright/upside-down × right/left) of the hand images were tested. The experimental session consisted of four blocks, as described above, and each block consisted of 120 trials (40 self hand trials and 80 other hand trials), in which the four image conditions were presented equally often; therefore, the total number of trials was 480. Before the test trials, each participant performed approximately 20 practice trials to determine whether they were performing the trials according to the instructions.

##### Task 2: Partner/Unknown Opposite-Sex Person Discrimination Task

The experimental procedure of Task 2 was the same as that used in the self–other discrimination task, except that the participants’ own hands and the same-sex others’ hands were replaced by their partners’ hands and an unknown opposite-sex persons’ hands.

### 2.3. Data Processing and Analysis

Accuracy and reaction time (RT) were recorded in each condition. For each participant, the RT outliers were removed by excluding trials with RTs that fell outside of 2 SD from the median of all trials. ANOVAs were conducted on accuracy and RTs with hand ownership (self/other or partner/unknown opposite-sex person), responding hand (right, left), hand image laterality (right, left), and visual perspective of hand image (upright, upside-down). The discriminability and response criterion (bias) for visual hand recognition were calculated as dprime and ln(β), respectively, according to signal detection theory (e.g., [31]). The dprime indicates how well the participant could discriminate the self (partner’s) image from the other’s (unknown person’s) image. As for ln(β), a positive ln(β) value indicates a bias toward others (unknowns), while a negative value indicates a bias toward self (partner).The calculation of these two values was based on the formula reported by Macmillan and Creelman [31]. These values were also entered into ANOVAs with three within-participant factors (i.e., responding hand [right, left], hand image laterality [right left], and visual perspective of hand image [upright, upside-down]). Bonferroni-corrected post-hoc comparisons were performed when necessary.

## 3. Results

### 3.1. Accuracy

#### 3.1.1. Task 1: Self-Other Discrimination Task

The overall accuracy was high (90%; see Table 1). A significant interaction between hand ownership and image laterality (*F*[1, 17] = 5.438, *p* = 0.0323, partial *η*^2^ = 0.2424) was found; however, a post-hoc comparison revealed that no significant differences were found. No other significant main effects or interactions were found (*p* > 0.05).

#### 3.1.2. Task 2: Partner/Unknown Opposite-Sex Person Discrimination Task

The overall accuracy was high (87%; see Table 2) and no significant main effects or interactions were found (*p* > 0.05).

### 3.2. RTs

#### 3.2.1. Task 1: Self-Other Discrimination Task

**Figure 2:**
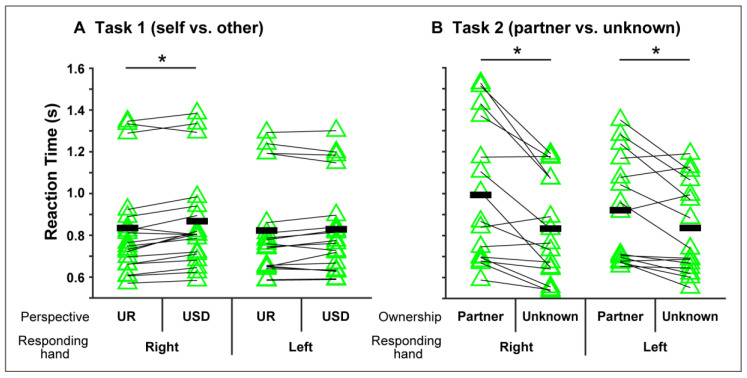
Mean reaction time (RT) in (**A**) Task 1 (self vs. other) and (**B**) Task 2 (partner vs. unknown). UR and USD denote upright and upside-down images, respectively. Black bars indicate mean values in each condition. * *p* < 0.05.

A main effect of hand ownership (*F*[1, 17] = 11.653, *p* = 0.0033, partial *η*^2^ = 0.4067; self: 0.881 ± 0.251 s; others: 0.797 ± 0.245 s) and a main effect of visual perspective of hand image (*F*[1, 17] = 7.655, *p* = 0.0132, partial *η*^2^ = 0.3105; upright: 0.829 ± 0.255 s; upside-down: 0.849 ± 0.247 s) were found, as well as an interaction between the responding hand and visual perspective (*F*[1, 17] = 13.122, *p* = 0.0021, partial *η*^2^ = 0.4356). A post-hoc comparison found a significantly longer RT in the upside-down image condition than in the upright image condition when the right hand was used for response (Figure 2A). No other significant main effects or interactions were found (*p* > 0.05).

#### 3.2.2. Task 2: Partner/Unknown Opposite-Sex Person Discrimination Task

As shown in Figure 2B, a main effect of hand ownership (*F*[1, 14] = 13.184, *p* = 0.0027, partial *η*^2^ = 0.4850; partner: 0.957 ± 0.313 s; unknowns: 0.834 ± 0.241 s) as well as an interaction between hand ownership and responding hand (*F*[1, 14] = 11.407, *p* = 0.0045, partial *η*^2^ = 0.4490) were found. A post-hoc comparison found a significantly longer RT in the partner condition than in the unknown condition when the responding hand condition was both right and left. No other significant main effects or interactions were found (*p* > 0.05).

### 3.3. Dprime and ln(β)

#### 3.3.1. Task 1: Self–Other Discrimination Task

For dprime, no significant main effects or interactions were found (*p* > 0.05), although a main effect of the visual perspective of hand images almost reached significance (*F*[1, 17] = 4.281, *p* = 0.0541).

**Figure 3:**
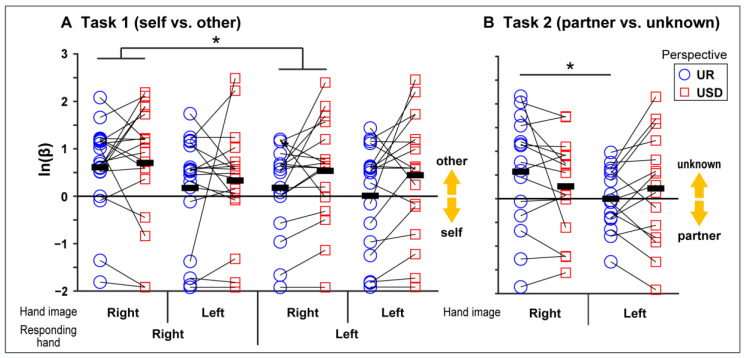
Mean ln(β) in (**A**) Task 1 (self vs. others) and (**B**) Task 2 (partner vs. unknown). UR and USD denote upright and upside-down images, respectively. Other main effects and interactions are described in the main text. Black bars indicate mean values in each condition. * *p* < 0.05.

As for ln(β) (response bias), a significant main effect of the laterality of hand images (*F*[1, 17] = 6.547, *p* = 0.0203, partial *η*^2^ = 0.2780) as well as a significant interaction between the responding hand and hand image laterality (*F*[1, 17] = 4.527, *p* = 0.0483, partial *η*^2^ = 0.2103) were found. A post-hoc comparison revealed a significantly smaller value of the left hand’s response than of the right hand’s response when a right hand image was presented. An interaction between the responding hand and visual perspective (*F*[1, 17] = 6.507, *p* = 0.0207, partial *η*^2^ = 0.2768) was also found. A post-hoc comparison found a significantly smaller value for the left hand’s response than for the right hand’s response when an upright image was presented (Figure 3A). No other significant main effects or interactions were found (*p* > 0.05).

#### 3.3.2. Task 2: Partner/Unknown Opposite-Sex Person Discrimination Task

For dprime, no significant main effects or interactions were found (*p* > 0.05).

As for ln(β), a main effect of responding hand (*F*[1, 14] = 18.594, *p* = 0.0007, partial *η*^2^ = 0.5705; right: 0.553 ± 1.130, left: −0.031 ± 1.096) was found. An interaction between visual perspective and hand image laterality (*F*[1, 14] = 5.163, *p* = 0.0394, partial *η*^2^ = 0.2694) was also revealed. A post-hoc comparison found a significantly smaller value for the left hand image than for the right hand image when the image was presented upright (Figure 3B).

## 4. Discussion

The current study explored how close relationships affect the performance of an explicit hand recognition task that involves comparing a partner’s hand with an unknown person’s hand. In addition to this comparison, participants in another group completed an explicit self hand recognition task to test whether the result of Ferri et al. [24] could be replicated. 

In Task 1, a longer RT was found for self hand image compared with images of another person’s hand. Although no significant difference in accuracy was found in Task 1, the current results are in accordance with the results of Ferri et al. [24]. Specifically, a “self-advantage” effect was not revealed when the participants were required to explicitly recognize hand identity. This result contrasts with the results of the hand laterality judgment task using self and other’s hand images (this task could be regarded as an implicit recognition task of hand ownership), in which the self-advantage effect emerged. Concerning the results of the self–other discrimination and hand laterality judgment tasks, previous studies [24,32,33] have suggested that motor simulation based on implicit sensorimotor knowledge about body parts (in this case, the hands), including a combined visuo-sensorimotor strategy [34,35], is necessary for the self-advantage effect. Indeed, Conson et al. [36] demonstrated that the self-advantage effect could emerge even in the explicit hand recognition task, when the task required a high “sensorimotor load” (see also [37]). Therefore, the cost of sensorimotor load when performing a task, rather than a dissociation between implicit versus explicit representations about self body, is an essential component in the identification of one’s own hand image. 

As described in the introduction section, the activation in the right EBA for the allocentric view of body parts is larger than that for the egocentric view [21,22], and this view difference (egocentric or allocentric) is an important cue for self–other discrimination (see also [38]). Conson et al. [25] performed a task similar to Task 1 in the present study, and their results showed an interaction between hand ownership, hand image laterality, and visual perspective, such as significantly faster RTs in the recognition of the other’s left hand image than in the recognition of the other’s right hand image when an upside-down image was presented, thereby confirming the association between hand ownership and visual perspective. Regarding visual perspective, the present study (Task 1) revealed (1) slower responses to the allocentric view of the hand image when responding with the right hand, and (2) the greater self-bias of the left hand’s response (in comparison with the right hand’s response) when the hand image was presented by the egocentric view. Although the reason why slower responses to the allocentric view emerged only when responding with the right hand (not with the left hand) will be clarified, the second result could be discussed from the association between self-recognition (favored by the egocentric view) and the right hemisphere, as described below.

Several studies have reported that hand responses are influenced by the controlling contralateral hemisphere when participants are required to respond to stimuli that are strongly lateralized in hemispheric processing [39,40,41]. Based on this evidence, Keenan et al. [42] performed a face-recognition task and found that self face stimuli were identified more rapidly than non-self face stimuli when the participants responded with their left hand, indicating that self-recognition may be correlated with activity in the right hemisphere. Although the current RT results were not in line with Keenan et al.’s [42] findings, the lower ln(β) when responding to the upright (egocentric) image with the left hand was close to zero (0.09) compared with the larger ln(β) in other conditions, where the response was biased toward the others (approximately 0.47). Of course, “close to zero” means neutral response bias and therefore cannot be described as a self-bias response. However, it could be argued that the response manner for the upright image with the left hand is relatively biased toward the self in comparison with other conditions, while the reason for the general response bias toward others in the current experiment requires further clarification. This finding suggests that the processing of self-recognition mediated by the right hemisphere was relatively strongly activated in the upright image (egocentric view) condition when responding with the left hand. However, a recent study by De Bellis et al. [43], using the same experimental paradigm of Ferri et al. [24] and the on-line high-frequency repetitive transcranial magnetic stimulation (rTMS) technique, revealed that the right EBA stimulation enhanced the implicit visual processing of others’ hands, whereas the stimulation of the left EBA led to a self-advantage. Therefore, how the left EBA interacts with other areas of the right hemisphere should be investigated more extensively in future studies. It should be noted that the primary difference in the experimental procedure used between Conson et al. [25] and the present study is that Conson et al. [25] used a foot response, whereas the present study employed a hand response. Whether and how the response manner (i.e., hand or foot) affects a hand recognition task must also be clarified. 

The main focus of the current experiment was on the effect of a close relationship (familiarity) on explicit hand recognition. Specifically, based on the “self-expansion (to the romantic partner)” account [26], focus was placed on whether the “partner-disadvantage” effect (in comparison with unknown persons), such as the self-disadvantage effect, emerges when performing explicit hand recognition. The results revealed longer RTs for images of a partner’s hand compared with images of another person’s hand, indicating slower responses to the partner’s hand image than to images of an unknown person’s hand, regardless of whether responses were made with the right or left hand. In the case of face recognition, Kircher et al. [44] argued that one’s own face is not processed differently on a behavioral level compared with another, over-learned, emotionally salient face (i.e., the partner’s face). The current results revealed longer RTs in the self and partner’s hand conditions compared with the other and unknown person’s hand conditions. As for the response bias, significantly smaller ln(β) values when responses were made with the left hand (−0.031) in comparison with the right hand (0.553) were also found in the partner/unknown opposite-sex person discrimination task (Task 2), indicating that participants’ responses were relatively biased toward the partner responding with the left hand in this experimental situation. The results revealing a bias toward the partner responding with the left hand (Task 2) and a bias toward the self for the upright image with the left hand (Task 1) indicate that the explicit hand recognition of the self and partner are similarly processed by the right hemisphere and mediated by the left hand. These results suggest that the explicit recognition processes of a romantic partner’s hand might be (at least partially) similar to those of one’s own hand, implying that self-expansion to a romantic partner could occur at explicit visual hand recognition.

The limitation of this study is the relatively small sample size, and therefore a future study designed by power analysis using the effect size obtained from the current study must be conducted. One remaining question is how the implicit hand recognition of partners is processed, so a hand laterality judgment task using the hand image of partners and unknowns should also be performed. In addition, the relationship length of each participant in Task 2 was relatively short because the data were collected from university students. Thus, future studies in which relationships (e.g., lover or spouse) and relationship lengths are included as independent variables are needed.

Overall, the current results indicate that the existence of a close relationship can induce the process of hand recognition when explicitly recognizing a partner’s to be similar to the process of explicitly recognizing one’s own hand. 

## 5. Conclusions

The current study focused on whether and how a close social relationship affects hand recognition and attempted to verify the results of previous studies (e.g., [24]) by investigating self hand recognition vs. the hand recognition of others. An experiment was performed in which the participants were required to identify the hand (of themselves or others in Task 1 and of a partner or unknown person in Task 2) in a situation in which the conditions of hand ownership, hand image laterality, and visual perspective of the hand image were manipulated. The results showed longer RTs to self and partner images than to images of others and unknowns, suggesting that close social relationships modulate hand recognition.

## Figures and Tables

**Table 1:** Mean accuracy (standard errors) of discrimination between self and other’s hand when responding with the right and left hands (Task 1).

	Upright				Upside-Down			
	Self		Other		Self		Other	
	Right	Left	Right	Left	Right	Left	Right	Left
Response with								
right hand	0.913	0.929	0.926	0.874	0.884	0.892	0.921	0.882
	(0.029)	(0.026)	(0.035)	(0.044)	(0.037)	(0.035)	(0.036)	(0.040)
left hand	0.923	0.956	0.908	0.877	0.891	0.890	0.909	0.886
	(0.040)	(0.013)	(0.034)	(0.039)	(0.032)	(0.037)	(0.035)	(0.039)

**Table 2:** Mean accuracy (standard errors) of discrimination between a partner’s and an unknown opposite-sex person’s hands when responding with the right and left hands (Task 2).

	Upright				Upside-Down			
	Self		Other		Self		Other	
	Right	Left	Right	Left	Right	Left	Right	Left
Response with								
right hand	0.819	0.850	0.902	0.869	0.839	0.823	0.853	0.862
	(0.040)	(0.036)	(0.039)	(0.040)	(0.042)	(0.042)	(0.054)	(0.041)
left hand	0.872	0.929	0.903	0.861	0.848	0.917	0.88	0.834
	(0.055)	(0.044)	(0.031)	(0.042)	(0.058)	(0.036)	(0.030)	(0.057)
